# Feasibility of Integrating Real-Time fMRI Neurofeedback with rTMS: Study Design, Preliminary Results, and Technical Challenges

**DOI:** 10.52294/001c.165511

**Published:** 2026-08-26

**Authors:** Lysianne Beynel, Vinai Roopchansingh, Paul Taylor, Richard Reynolds, Linqing Li, Neil Baker, Daryl Bandy, Katherine Cameron, Zhi-De Deng, Ekaete Ekpo, Hannah Gura, Sahit Menon, Eva Wiener, Zeynab Rezaee, Justin Rajendra, Bruce Luber, Sarah H. Lisanby

**Affiliations:** 1 National Institutes of Health,; 2 Howard University,; 3 University of Pennsylvania,; 4 Vanderbilt University Medical Center,; 5 Johns Hopkins University,; 6 Arizona State University

**Keywords:** rTMS, real-time fMRI neurofeedback, amygdala

## Abstract

Repetitive transcranial magnetic stimulation (rTMS) is a noninvasive brain stimulation technique used in the treatment of several psychiatric disorders. However, its clinical efficacy remains limited in some cases, in part due to the lack of control over ongoing brain state during stimulation. Although some studies have combined rTMS with psychotherapy to engage the brain state during stimulation, brain state is still not objectively measured in such studies. Real-time functional magnetic resonance imaging neurofeedback (rt-fMRI-nf) offers a promising approach to objectively monitor and modulate brain state during rTMS. Here, we report the first study to investigate the feasibility of combining connectivity-based rTMS with rt-fMRI-nf to modulate amygdala activity. First, participants completed an fMRI emotion-matching task to identify an individualized medial prefrontal cortex (mPFC) target showing the strongest negative functional connectivity with the right amygdala. Across four subsequent visits, participants received active rTMS to either a mPFC target or to the vertex (control site) while engaged in rt-fMRI-nf. During rt-fMRI-nf, participants were presented with aversive images and were instructed either to passively look at them or to downregulate the height of a visual gauge representing their amygdala activity. We expected that rTMS during this downregulation would decrease amygdala activation even more than during downregulation alone. Feasibility, signal quality, rTMS-induced artifacts, and participant experience were systematically evaluated. We demonstrate that while concurrent connectivity-based rTMS and rt-fMRI-nf is feasible, the integration presents substantial technical and methodological challenges. These include limitations related to RF coil selection, signal-to-noise ratio in deep brain structures, prolonged rTMS-induced artifacts, and inter-individual variability in amygdala engagement. Despite these challenges, this work establishes a methodological foundation for future studies integrating real-time neuroimaging and neuromodulation. Continued advances in hardware, acquisition strategies, and individualized targeting may enable more precise state-dependent stimulation of emotion-related circuits, with potential implications for precision interventions in psychiatric disorders.

## INTRODUCTION

Repetitive transcranial magnetic stimulation (rTMS) is a noninvasive neuromodulation technique that has been FDA-approved as a treatment for major depressive disorder, smoking cessation, migraine, anxiety in depression, and has the potential to treat a variety of other psychiatric disorders such as post-traumatic stress disorder (PTSD). While widely used, rTMS efficacy remains limited for some patients. For example, a recent meta-analysis of randomized, sham-controlled trials in adults with major depressive disorder reported clinical response rates of approximately 40%, indicating that nearly 60% of patients do not meet conventional response criteria following a standard course of rTMS.^1^ Several factors likely contribute to the low response rate including targeting approach and stimulation intensity; another factor that is likely to contribute but largely ignored is the lack of control of brain state during stimulation. The large variability of subjective brain states during rTMS can dramatically influence the directionality and strength of rTMS effects.^2^ One way to control for state-dependent effects is to apply rTMS while engaging the targeted brain network during stimulation. When properly targeted and timed, online rTMS during a task can enhance cognitive functioning,^3,4^ but results can vary due to additional factors such as dosing, targeting, and age.^5–8^ Recent work combining cognitive behavioral therapy with rTMS has shown promising results for major depressive disorders.^9,10^ Similarly, the combination of rTMS with exposure-based psychotherapy for patients with PTSD has been explored and shown to be effective, though conferring only a moderate additional clinical benefit.^11,12^

While these combined approaches seem to improve rTMS efficacy, recent meta-analyses (e.g.^13^) demonstrate that the clinical benefit resulting from this combination is still moderate. A potential explanation for this limited benefit could stem from the lack of an objective measurement of how well brain state is being maintained during the manipulation. Notably, none of these studies have directly measured brain state during stimulation or conducted ongoing or individualized assessments of success. Without this information, it is challenging to know whether the stimulated network was successfully engaged during the therapy. Real-time functional magnetic resonance imaging neurofeedback (rt-fMRI-nf) could help fill this gap by providing objective dynamic information about ongoing brain state, allowing for individualized stimulation paradigms. To our knowledge, there are no existing reports of research combining rt-fMRI-nf with rTMS to date.

In this study we aimed to evaluate the feasibility of combining connectivity-based rTMS and rt-fMRI-nf to decrease amygdala activity. Given the amygdala’s critical role in a variety of psychiatric disorders, we sought to apply active rTMS to either the location within the medial prefrontal cortex (mPFC) with the strongest negative connectivity with the right amygdala or to the vertex (control site). Individualized stimulation targets were defined after participants completed a first visit during which they performed an emotion matching task^14^ that is known to elicit amygdala activation. Then during four subsequent visits, participants received rTMS to the vertex or to the mPFC (two visits per stimulation site) while engaged in rt-fMRI-nf. During rt-fMRI-nf, participants were presented with aversive pictures and asked to either look passively at them or to downregulate their amygdala activity while viewing them. At the end of the last visit, participants were asked to perform a short memory retrieval task to ensure they looked at the pictures, followed by a debriefing session to assess the study’s feasibility and gather feedback on their experience.

This manuscript first provides all the required technical steps to combine rt-fMRI-nf and rTMS, then details the methods and preliminary results of the rt-fMRI-nf and rTMS project. It also describes and demonstrates many of the methodological challenges associated with this pairing: rTMS coil positioning, multi-echo acquisition, rTMS artifact and impact on the neurofeedback signal, low signal-to-noise ratio and provides potential solutions for some of them. These challenges present opportunities for future technical development to advance methodology for precision brain state manipulation and modulation.

## METHODS

### 1. REQUIRED TECHNICAL STEPS TO PERFORM RT-FMRI-NF COMBINED WITH RTMS.

#### 1.1. HARDWARE

##### 1.1.1. CONNECTING THE EQUIPMENT

The scanner console was connected to a Linux computer, on which AFNI was installed, via ethernet. The scanner vendor’s real-time DICOM export functionality wrote the MRI DICOM images directly to the Linux computer. The experimental computer, which displayed the stimuli, and computed and displayed the neurofeedback signal to participants, was also connected to the real time computer via ethernet, and the IP address of the experimental computer was set in AFNI’s real-time module. The experimental computer also used a Current Designs interface unit, which received a TTL pulse from the scanner at the start of each TR. The button box translated each of these pulses to a ‘t’ key press for each TR. A counter of those pulses was used to build the timing of the experiment.

##### 1.1.2. DEVELOP A HEAD AND MIRROR HOLDER

**Figure 1.**
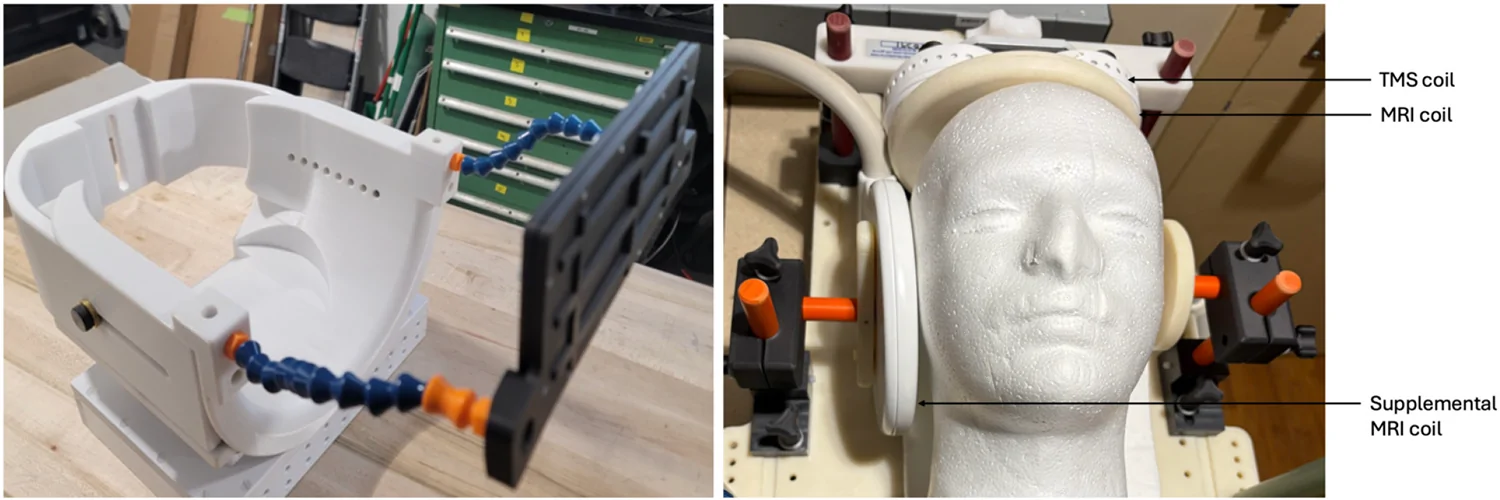
First (left) and second version (right) of the head holder

This experiment uses two 7-channel MRI coils which allowed to apply TMS during MRI acquisition while avoiding the limitations of a classic birdcage coil, such as the restricted space that makes optimal TMS-coil positioning difficult. However, there are consequences to the absence of the bird cage receiver coil; specifically, we had to address the lack of a structure to hold the mirror (on which the subject views stimuli) and a lack of support for padding to limit head motion. NIMH’s Section on Instrumentation developed a head holder that allowed us to position the supplemental 7-channel coil behind the participant’s head and to place a mirror so that participants could see the screen behind them (Figure 1, **left**). However, after piloting we noticed that the signal to noise ratio in the right amygdala was low, and that positioning the supplemental coil on the side of the head increased the signal; therefore, we used the second and final version of the head holder (Figure 1, **right**).

##### 1.1.3. TRIGGER THE RTMS PULSE DURING MRI ACQUISITION

A Psychopy script read TTL pulses from the scanner (via the Current Designs interface unit) to display each stimulus of the task (instruction, images, and fixation cross) and sent the rTMS pulse. To do so we used a data acquisition and control product (LabJack U3, LabJack Corporation, Lakewood, CO, USA) that was connected to the experimental computer and the rTMS stimulator. When the third image is presented and in 50% of the trials, the LabJack device sent a pulse to the TMS machine to trigger the rTMS sequence. This device introduces a USB communication latency on the order of approximately 0.5–1 ms for transmission of a digital I/O command from the host computer to the device. Following receipt of the command, the device processing time is in the microsecond range before execution of the output pulse. Therefore, the total trigger-related delay introduced by the LabJack U3 is expected to be in the low millisecond range (approximately 1–2 ms in total). However, after measuring the actual time between the MRI scanner TTL pulse and the delivered TMS pulse we found a delay of 22 ms, this additional time is probably due to the cumulative latency of the entire triggering chain, including MRI scanner TTL generation, USB communication and buffering in the LabJack U3, software scheduling within the stimulus/control computer operating system, and the intrinsic response latency of the TMS device itself; but is still negligible relative to the temporal resolution of fMRI acquisition and the hemodynamic response function.

#### 1.2. SOFTWARE: SETTING UP AFNI REAL TIME

As the EPI images were recorded with a multi echo sequence (**see** section 2.3.2. RT-fMRI NEUROFEEDBACK PARADIGM), we set up some specificity to account for this. The DICOM images were sorted (scanner-specific) by AFNI’s *Dimon*, which sent one volume per echo and time point to AFNI’s real-time plugin, using the following settings: the registration was set at “3D:real time”, resampling was set at “Quintic”; the mask was the Amygdala in EPI space generated by the alignment script; the Vals to Send were defined as the “ROI means”. The registration base of the ongoing EPI data was an *external dataset* defined as the minimum outlier volume from echo 2 of the 20 volumes of EPI acquisition “epi_base_MO” (the last 16 volumes). This file was generated by the short *afni_proc.py* script (**see** section 3.2.1. PRE-PROCESSING: CONVERT THE AMYGDALA MASK FROM MNI SPACE TO EPI SPACE) with the base image for this volume left at 0. Registration of the echoes was done by setting the plugin’s MergeRegister to “reg channels”, performing the registration based on the second echo (source channel = 1, as this is 0-based), which then applied the same alignment transformation to all echoes. The two echoes were then merged using the optimally combined approach, setting Channel merge to “Opt Comb”. This option required a T2* reference data set, which along with the external registration base volume was generated using the previously mentioned short *afni_proc.py* command to optimally combine the initial 20 volume EPI acquisition. These options were configured in a *demo.2.afni.tcsh* script.

### 2. EXPERIMENTAL PROCEDURE

**Figure 2.**
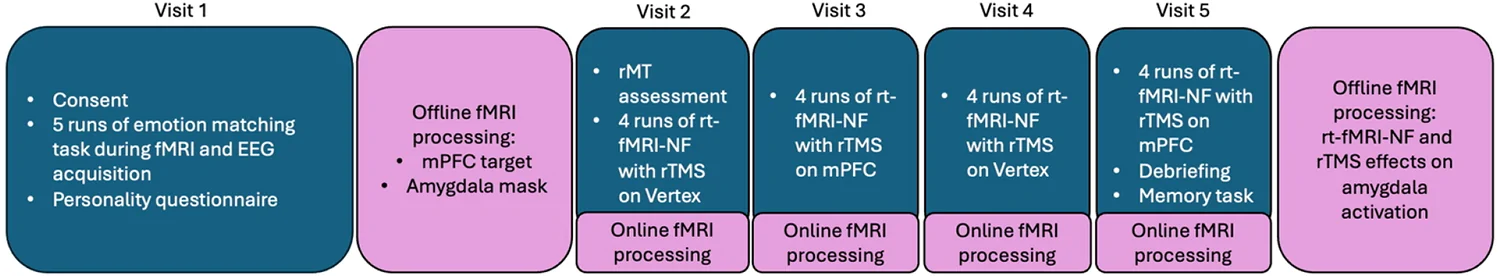
Illustration of the experimental protocol

Our protocol involved five visits (Figure 2). During the first visit, participants provided written informed consent for our protocol (registered at clinicaltrials.gov as NCT03351764, sub-study 3), then completed five runs an emotion matching task^14^ during simultaneous fMRI and EEG acquisition. They also completed temperament and personality questionnaires; however, EEG and questionnaire data are not presented in this manuscript. fMRI data from this first visit were used as a functional localizer to generate an individualized amygdala mask based on BOLD activation, and to identify a mPFC rTMS target with strong connectivity to the amygdala, using a psychophysiological interaction (PPI) analysis. Participants came back for four subsequent visits during which they performed the real-time fMRI neurofeedback task. During this task, they were presented with aversive pictures and asked to either look at them without trying anything to modify the bar height or to try to decrease the bar height (i.e., downregulate their amygdala activation). rTMS occurred during half of the trials at each visit, targeting either on the mPFC or on the vertex (control site). During the first visit, hotspot and resting motor threshold (rMT) were assessed to define the stimulation intensity for the subsequent visits. During the last visit, participants completed a debriefing questionnaire to assess their subjective experience about the experiment, as well as a recognition memory task to ensure they paid attention to the pictures during the experiment.

#### 2.1. PARTICIPANTS

The four participants included in this feasibility study, two males (32 and 34 years old) and two females (23 and 33 years old), provided written informed consent prior to study participation. Subjects were screened for and had none of the following exclusion criteria: currently pregnant or breastfeeding, history of any Axis I DSM-5 disorder, history of seizure, history of epilepsy in self or first degree relatives, stroke, brain surgery, known structural brain lesion, history of any head trauma, increased risk of seizure for any reason, or currently taking medication that lowers the seizure threshold, history of drug or alcohol abuse within one year or a lifetime history of drug or alcohol dependence, presence of ferromagnetic metal in the body, unstable or serious medical or neurological disorder, were HIV positive, or had hearing loss that had been clinically evaluated and diagnosed.

#### 2.2. VISIT 1: EMOTION MATCHING TASK AND MRI ACQUISITION

Participants were scanned using a 3T MRI (Siemens Skyra) scanner equipped with a 64-channel head coil. An anatomical T1-weighted volume was acquired (TR = 2530 ms, TE=3.3 ms, voxel size = 1 × 1 × 1 mm, FOV = 256 mm). Five fMRI runs of multi-echo EPI were then collected while participants were performing the face-matching emotion task^14^ (TR = 2000 ms, TE1 = 12.6 ms, TE2 = 30.22 ms, TE3 = 47.84 ms; multiband acceleration factor = 2; voxel size = 3 × 3 × 3 mm, FOV = 192 mm; flip angle = 60 degrees, this sequence was a research pulse sequence derived from the Center for Magnetic Resonance Research sequence^15^ and adapted to meet the specific requirements of our protocol. The flip angle of 60 degrees was chosen as in the fMRI acquisitions in this work, signal fluctuations are largely dominated by physiological noise (e.g., respiration and cardiac effects), rather than thermal noise. As a result, using a flip angle in this range provides several practical advantages (and increasing it would not be expected to increase TSNR): it reduces specific absorption rate (SAR), improves tissue contrast stability over time, and can enhance robustness to motion and registration errors across volume.^16^ These factors are particularly important for longitudinal signal consistency and downstream analyses. Therefore, a flip angle of 60° represents a balanced choice between signal strength, physiological noise considerations, and data quality stability.

**Figure 3.**
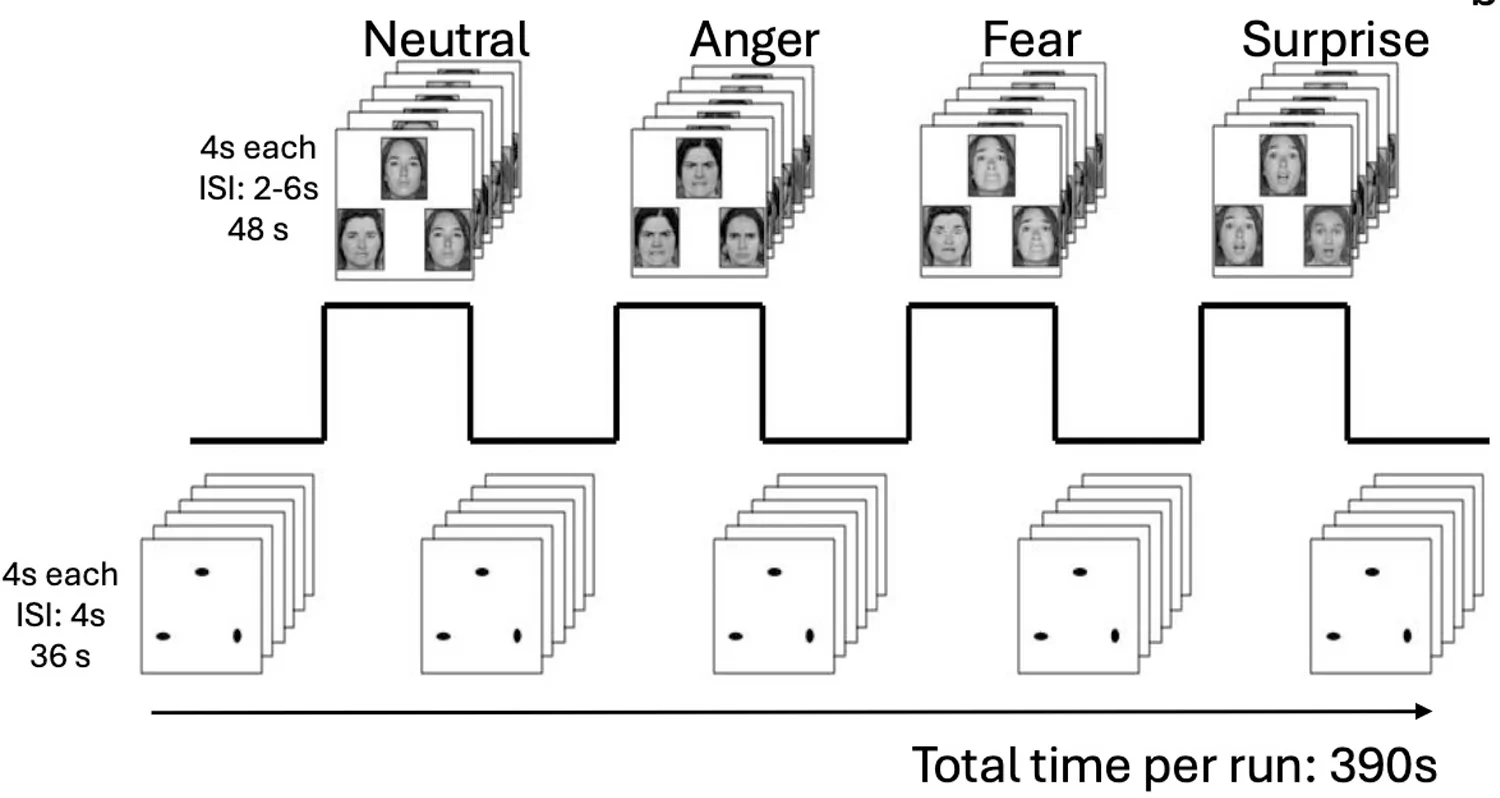
Illustration of one run of the emotion matching task.

Each run of the face-matching emotion task consisted of four blocks of the task (requiring participants to select the face that matched the probe), and five blocks of a shape-matching task (requiring participants to select the shape that matched the probe). In each block, 6 trios of faces or shapes were presented with an interstimulus interval (ISI) varying between 2–6 seconds for the face trials to reduce habituation; and a fixed ISI of 4 seconds for the shapes (Figure 3). Each block was separated by an instruction screen displayed for 2 seconds. Each run lasted 390 seconds, resulting in a total duration of 32.5 minutes across all runs. Stimuli were rear projected onto a screen that subjects viewed via a mirror system located on the head coil. The start of each run was synchronized with the MRI acquisition using the TTL signal, a digital electric signal generated by the EPI sequence that switches between two voltage levels, that allows to trigger events. Throughout each run, TTL pulses were also recorded at the start of each volume. Accuracy and reaction time were collected for each trial.

#### 2.3. VISITS 2–5: RTMS DURING REAL-TIME FMRI NEUROFEEDBACK

##### 2.3.1. RTMS

Resting motor threshold (rMT) was assessed at the beginning of the second visit, outside the scanner with the rTMS coil mounted on the 7-channel coil. For all participants, the rMT was quite high (NF001 = 90% MSO, NF002 = 84% MSO; NF003 = 90% MSO; NF004 = 93% MSO) due to the increased distance between the coil and the scalp. Since magnetoencephalography studies have demonstrated that in response to threatening stimuli, activation changes in the amygdala were found in the theta range (4–7 Hz)^17^; and since notable cortical-amygdala interactions occur in the theta range,^18^ rTMS was applied in 5Hz bursts at 120% resting motor threshold, for 4 seconds (20 pulses, replicating the parameters used by^19^ and^20^ (**see** section 4.3.1. RTMS COIL OVERHEATING). rTMS was then applied in half of the trials which will allow testing whether our neurofeedback paradigm described below could induce amygdala activation when presenting aversive images and whether participants were able to downregulate this activation without rTMS.

##### 2.3.2. RT-FMRI NEUROFEEDBACK PARADIGM

**Figure 4.**
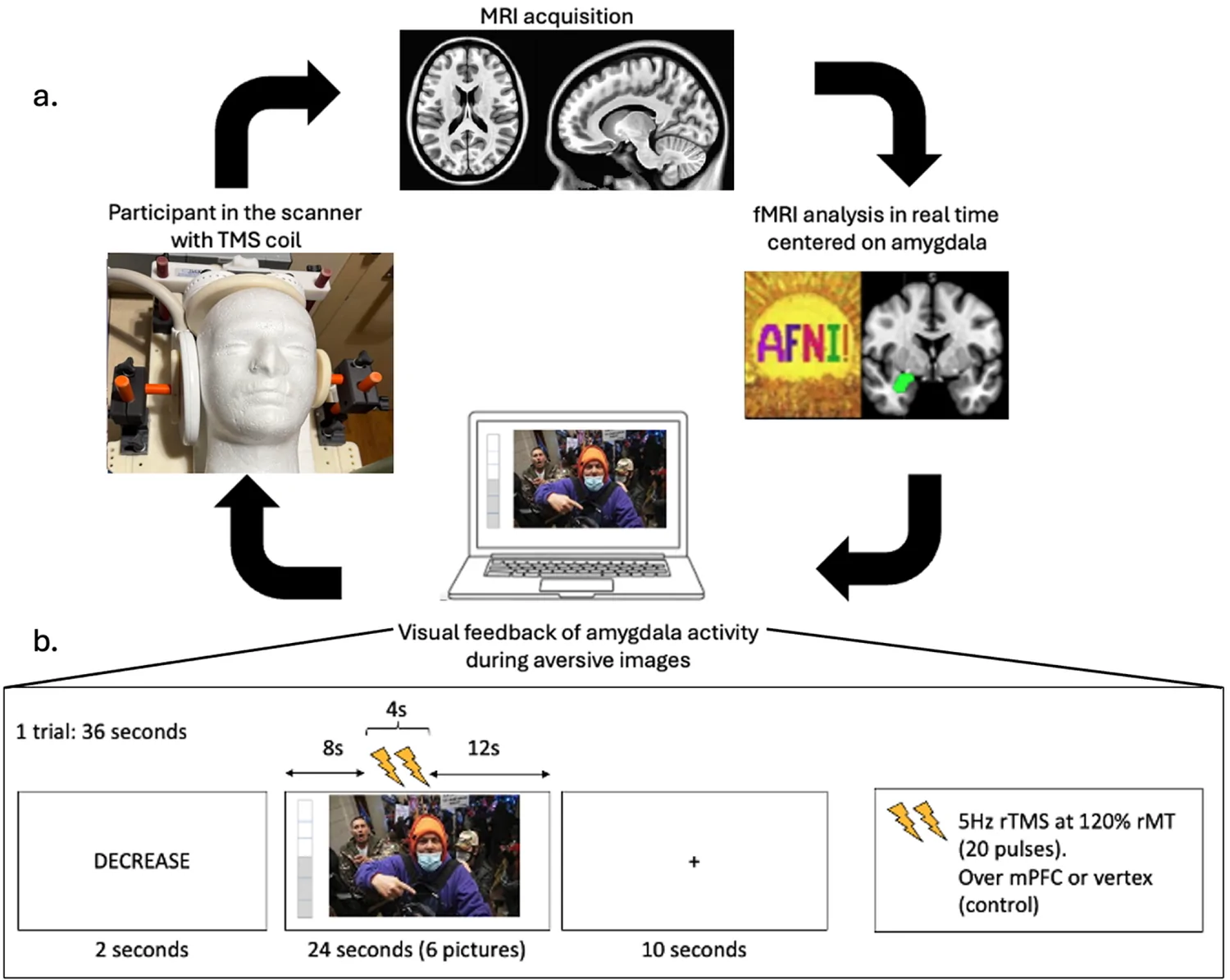
**a.** Illustration of rt-fMRI neurofeedback setup with 7-channel MRI coil. **b.** Illustration of one trial with rTMS timing. In conditions using rTMS (every other trial), short trains of rTMS were applied 8 seconds after the initial picture onset, to coincide with the onset of the third picture presentation in the series, with the train ending with the end of the third picture presentation, providing enough time for the participants to actively control their brain state.

For this procedure, instead of using the standard 64-channel head coil, participants were scanned with two 7-channel MRI coils^21^ (Alsix, Vienna; Magventure Denmark) (see Figure 4a). After installing the participant in the scanner and positioning the rTMS coil on the vertex or on the mPFC depending on the visit **(see** section 4.3.3. LIMITS TO RTMS COIL POSITIONING WITHIN A 7-CHANNEL MRI COIL), a T1-weighted scan was collected (same parameters as in visit 1), followed by a 20-volume multi-echo EPI scan (TR = 2000 ms, TE1 = 14 ms, TE2 = 33 ms; voxel size = 3.75 × 3,75 × 3,75 mm, FOV = 240 mm). Note that we reduced the number of echoes from three (used in the first visit) to two after noticing during piloting that the computer could no longer reconstruct the fMRI data in real time with three echoes, as the third echo introduced excessive processing delays (**see** section 4.3.2. LIMITS TO MULTI ECHO ACQUISITION DURING NEUROFEEDBACK). The same parameters were used for the rt-fMRI-nf acquisition, with 370 volumes per run.

The neurofeedback task began with a 2-second instruction screen telling participants to either “Decrease” the height of the bar for the neurofeedback condition (60 % of the trials in each block) or to ‘View’ for the control condition (40% of the trials). Participants were then shown a series of six aversive images drawn from the International Affective Picture System,^22^ the Geneva Affective Picture Database (GAPED),^23^ and the Necki Affective Picture System (NAPS)^24^ each shown for 4 seconds, for a total of 24 seconds per trial. A multi-image trial was chosen over a single image for the full 24 seconds to minimize amygdala habituation that may occur over that time, similar to the parameters used in,^25^ and as highlighted by our recent paper,^26^ in which we demonstrated using skin conductance recordings that presenting blocks of six aversive images every four seconds yielded sustained skin conductance level with small increases following each image onset, compared to presenting blocks of one image for 24 seconds, elicited a sharper initial peak, but the response decayed more rapidly. Aversive (Valence < 3.5 IAPS & NAPS, <35 GAPED) and highly arousing (Arousal > 5.5 IAPS & NAPS, 55 GAPED) images were presented from six categories: snake & bug, animals, human single, humans interacting, gore, and object/scene, as defined by the experimenter. Images were evenly distributed across trials so that each trial has one image from each category. A fixation cross was then presented for 10 seconds to allow their hemodynamic response to return to its baseline level before receiving instructions for the next trial (Figure 4b). There were 20 trials within each run (~13 minutes) and 4 runs per visit.

### 3. FMRI ANALYSES

#### 3.1. OFFLINE FMRI PROCESSING ANALYSIS AFTER VISIT 1

The BOLD (blood oxygen dependent) fMRI data were first analyzed to identify an individualized mask within the right amygdala using the Faces - Shapes contrast. This mask was then used for two purposes: (1) to conduct a psychophysiological interaction (PPI) analysis and identify the region within the medial prefrontal cortex (mPFC) showing the strongest negative connectivity with the right amygdala—which was subsequently used as the rTMS target; and (2) to extract the signal for real-time fMRI neurofeedback. All analyses were performed using the AFNI^27^ suite of tools, version 24.0.08.

##### 3.1.1. BOLD ANALYSIS

First, skull stripping and estimating nonlinear alignment (via *sswarper2)*^28^ of the anatomical image from native space to Montreal Neurological Institute (MNI) stereotaxic space was performed. Then, *afni_proc.py*^29^ was used to set up a full pipeline for the fMRI analysis of each participant, including the automatic generation of a quality control HTML for evaluating the data and processing steps.^28,30^ The first four TRs (8 seconds) of each run were excluded from analysis to allow stabilization of the measured signal. All images were corrected for slice acquisition timing, motion corrected by registration to the minimum outlier volume, and spatially smoothed with a 4 mm full-width-half-maximum smoothing kernel. EPI-anatomical alignment was be performed using the lpc+ZZ cost function with local EPI unifizing for additional stability, and these datasets were checked for left/right consistency.^31^ To reduce effects of participant motion, volumes with large motion (Euclidean norm, Enorm > 0.3 mm) between successive time points were censored. The multiple EPI echoes were processed withing *afni_proc.py* using *tedana*,^32^ with the ICA denoising based on the estimated BOLD decay across echoes and the temporal variance of the combined time series.

**Figure 5.**
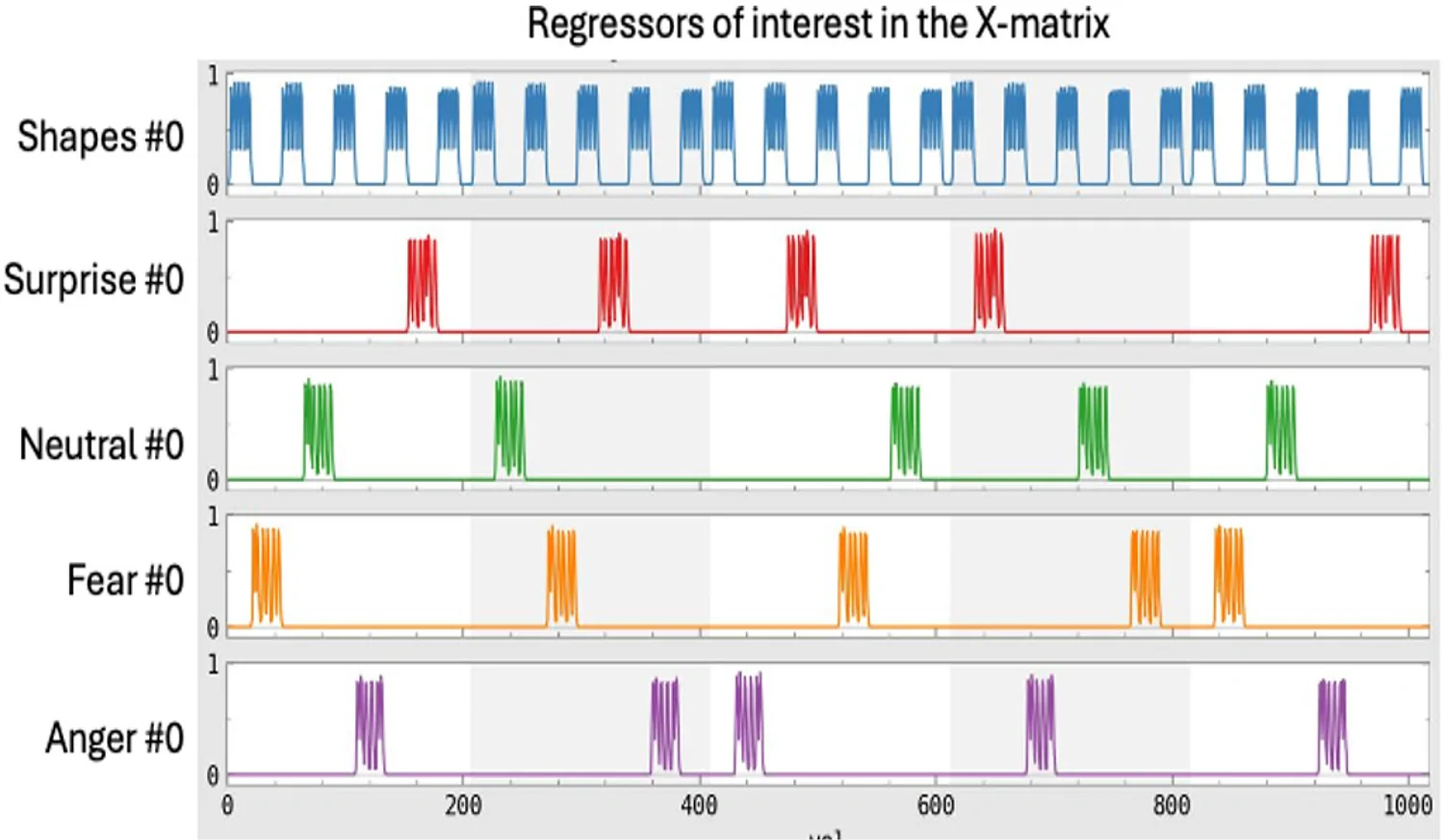
Task regressors used for fMRI processing.

For the regression modeling within *afni_proc.py*, given the fast response of the amygdala, a GAM regression basis, which assumes an instantaneous hemodynamic response, was used. Separate events were modeled for each emotional face of the face matching task (Anger, Fear, Neutral, Surprise) and for each shape of the shape matching block (Figure 5). The contrast between Emotional Faces - Shapes (Emotional Faces excluded the neutral condition) was generated. After regression modeling, several steps were then performed for QC evaluation using the APQC HTML, including checking the alignment between the anatomical, EPI, and the template image, checking that the stimuli were properly assigned between each stimulus class, and checking how many data points were censored because of motion.

Given that the emotion matching task (i.e. viewing Emotional Faces and Shapes) resulted in different sites of peak activation for each participant (Figure 8a), we decided to use their own activation as a mask for (1) subsequent PPI analysis, and (2) as a mask to extract the signal for real-time fMRI neurofeedback.

To create the individualized region mask on the final EPI data in template space, we first downloaded an amygdala ROI from Neurosynth (https://neurosynth.org/), extracted only the right hemisphere portion, and applied a threshold (15 < z < 33) using *3dcalc* to obtain a large ROI centered on the right amygdala. This ROI was then resampled to match the resolution of the statistical map using *3dresample*. Next, within this mask, we identified the top 25% most significantly activated voxels in the Faces - Shapes contrast. To do so, we used *3dBrickStat* to determine the t-score threshold corresponding to the top 25% of significantly activated voxels (p < 0.05) and then applied 3dcalc to retain only voxels exceeding this threshold. The resulting set of voxels constituted the individualized right amygdala mask (Figure 8b).

##### 3.1.2. PPI ANALYSIS

The second analysis consisted of a psychophysiological interaction (PPI) analysis conducted to characterize the functional connectivity between the right amygdala—significantly activated during the emotion-matching task—and the rest of the brain. The objective was to identify the region within the medial prefrontal cortex (mPFC) that exhibited the strongest negative connectivity with the amygdala. This spot was then used as the individualized stimulation target for connectivity-based rTMS, with the goal of indirectly modulating amygdala activity through its functional connectivity with the mPFC^33^.

To perform the PPI analysis, we used the individualized top 25% right amygdala mask, as described above, then *3dTproject* was run to orthogonalize the time series with respect to the main *afni_proc.py* analysis components, but without censoring, followed by *3dmaskave* to extract the average amygdala time series. A script was then used to partition this right amygdala time series based on stimuli presentation and convolve them into task-specific regressors, also using a GAM basis function. A final regression was then run to generate the PPI: Faces - Shapes contrast. As shown in Figure 8c we were able to find a negative connectivity between the right amygdala and the mPFC for each participant. The statistical map of this contrast was then converted from MNI space to native space using *3dNwarpCat* and *3dNwarpApply* commands. The statistical map in native space and the subject anatomical MRI were then loaded onto our neuronavigation software (Brainsight, Rogue Research, Canada) and the peak negative connectivity within the mPFC was defined as the rTMS target.

#### 3.2. ONLINE FMRI PROCESSING DURING VISIT 2–5

##### 3.2.1. PRE-PROCESSING : CONVERT THE AMYGDALA MASK FROM MNI SPACE TO EPI SPACE

**Figure 6.**
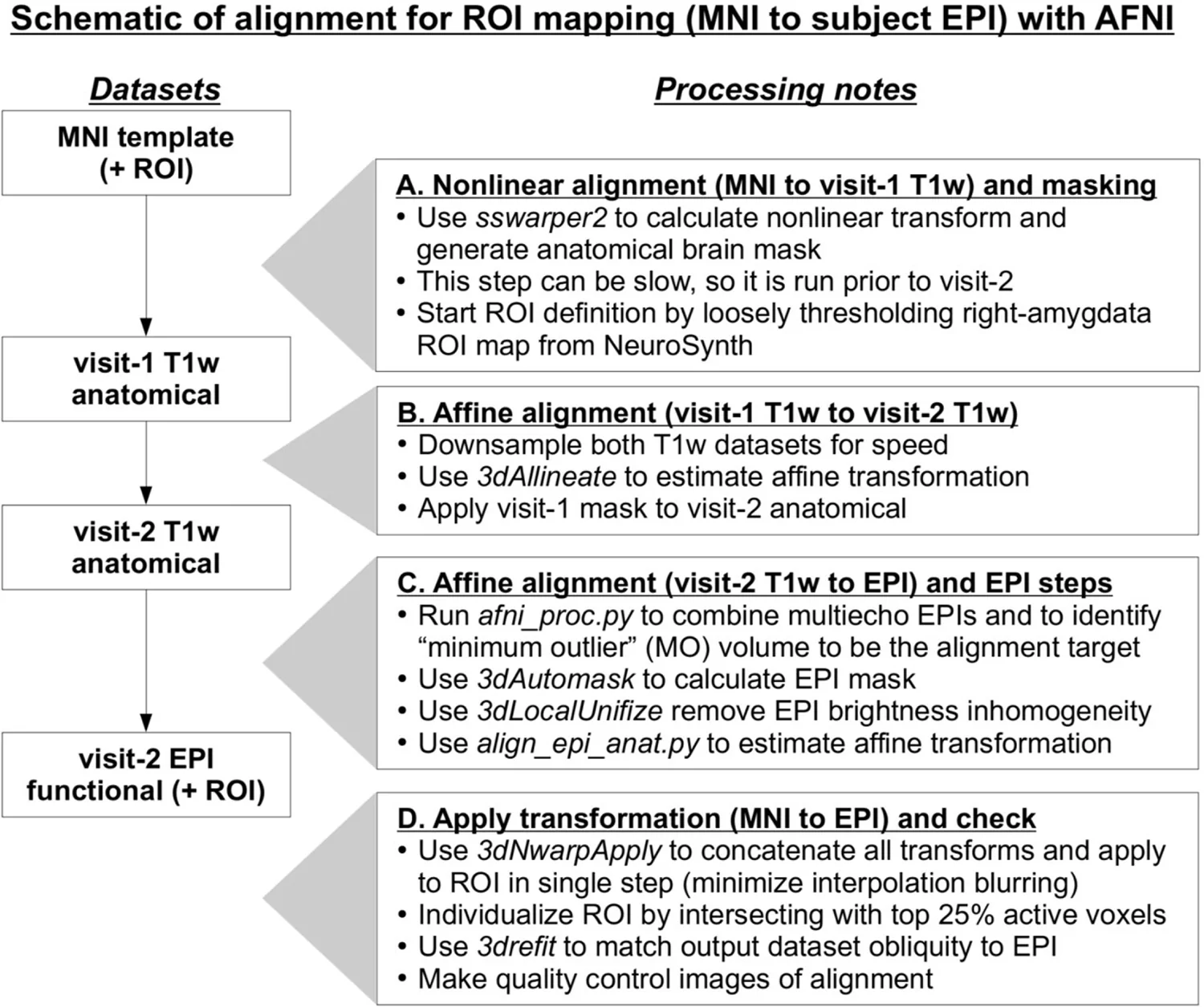
A schematic of alignment and supplementary processing steps performed with AFNI to map the region of interest (right amygdala downloaded from Neurosynth) from MNI standard space to the subject’s EPI grid. The nonlinear alignment in A is performed prior to the second visit. The remaining steps are calculated in a pair of scripts when the second visit is in progress, so the commands are focused around estimating high quality alignment in a minimal amount of time. A brief afni_proc.py command^29^ is run during C to combine the EPI volumes across echos using optimal combination (OC),^34^ and to identify the volume with the minimum number of outliers in a brain mask, making it the most reliable to serve as a target for alignment.

A set of processing scripts was run to map the individualized right amygdala mask from MNI space to the subject EPI grid, for the purpose of extracting signal there during the fMRI acquisition. Fig. 6 shows a schematic of the procedure, using programs within AFNI. The full set of steps is performed over two stages. First, after visit-1 but prior to visit-2, step A in Fig. 6 was performed, with nonlinear alignment from the MNI space to the subject’s anatomical space using *sswarper2*. In general, sswarper2 is too time and computationally intensive to be run during the second visit, hence it is done ahead of time. This processing estimates the nonlinear warp between the two datasets and produces a mask of the T1w dataset’s brain.

Next, steps B-D in Figure 6 occurred during the participant’s second visit, after another T1w anatomical and an initial 20 volumes of EPI dataset had been acquired. The scripts (available in the project’s GitHub repository: https://github.com/afni/aproject_realtime_fmri_amygdala) were developed to run as quickly as possible while also providing high quality alignment of the subject data. In step B, *3dAllineate* was used to estimate an affine transform between the visit-1 and visit-2 anatomical datasets, which have been upsampled in place to speed up processing time. After this alignment, the visit-1’s anatomical mask were applied to the visit-2 anatomical at full resolution, for efficient skull-stripping. In step C, a very short *afni_proc.py* command was run to perform optimal combination (OC) on the multi-echo EPIs, leaving one volume per time point. Additionally, the command also identified the EPI “minimum outlier” volume from echo 2, i.e., the time point with the fewest fraction of outliers in the brain mask, which served as the representative alignment target for the time series. After running *3dAutomask* on the EPI dataset, it was aligned to its concurrent visit-2 anatomical using *align_epi_anat.py*. Since all the necessary alignments have all been performed, in step D their full set of transforms were concatenated and applied to map the MNI-space ROI to the subject EPI grid, with minimal smoothing from interpolation. A quality control (QC) image was automatically generated with *@chauffeur_afni* to facilitate evaluating the results.

##### 3.2.2. ONLINE DATA ANALYSIS FOR RT-FMRI NEUROFEEDBACK SIGNAL

The rt-fMRI-nf signal was displayed as a grey 10-point gauge on the left side of the picture and represented the activity within the individualized mask in the right amygdala. The right amygdala was selected over the left since previous rt-fMRI neurofeedback studies have shown stronger right amygdala activation during emotional downregulation.^35^ It was suggested that this may be due to stronger involvement of the right amygdala in the automatic processing of emotions, with the left side more involved in cognitive processing.^36^

To compute the signal in the right amygdala, we used *3dDeconvolve* to generate an ideal hemodynamic response using a BLOCK regression model with a 24-second duration to match the image presentation, only during the View trials; we also added a linear drift correction. A regression was then computed between the ideal response and the current acquired data. The first element of this regression, the intercept, was defined as the baseline beta weight, while the second element represented the linear drift. The third beta weight represented the magnitude of the response for the View trial. For the first trial, which was always View, the bar gauge was set at 5. Participants were instructed that the gauge would not vary during this trial, as it was being used to compute the baseline value. We then computed the change in amygdala activation as the difference between the BOLD signal at the current TR and the baseline beta weight, divided by the View beta weight. We expected the change to vary between 0 and 1 and therefore to compute the gauge, we multiplied the change by 10 and added 0.5 to round it to the nearest whole number and convert it to an integer and convert it to a level on the gauge. However, during piloting sessions on team members, we noticed that the bar was moving too quickly from 0 to 10. Therefore, we added a comparison to the previous bar height so that the current change in bar height was computed as the signed square root of the change, which allowed us to smooth the changes. Finally, a last additional step had to be added because of the rTMS pulse, which created a large artifact on the fMRI data; thus, for trials with rTMS (every other trial) we had to remove the 2 rTMS TRs from the regression model.

#### 3.3. OFFLINE FMRI PROCESSING AFTER VISIT 2–5

**Figure 7.**
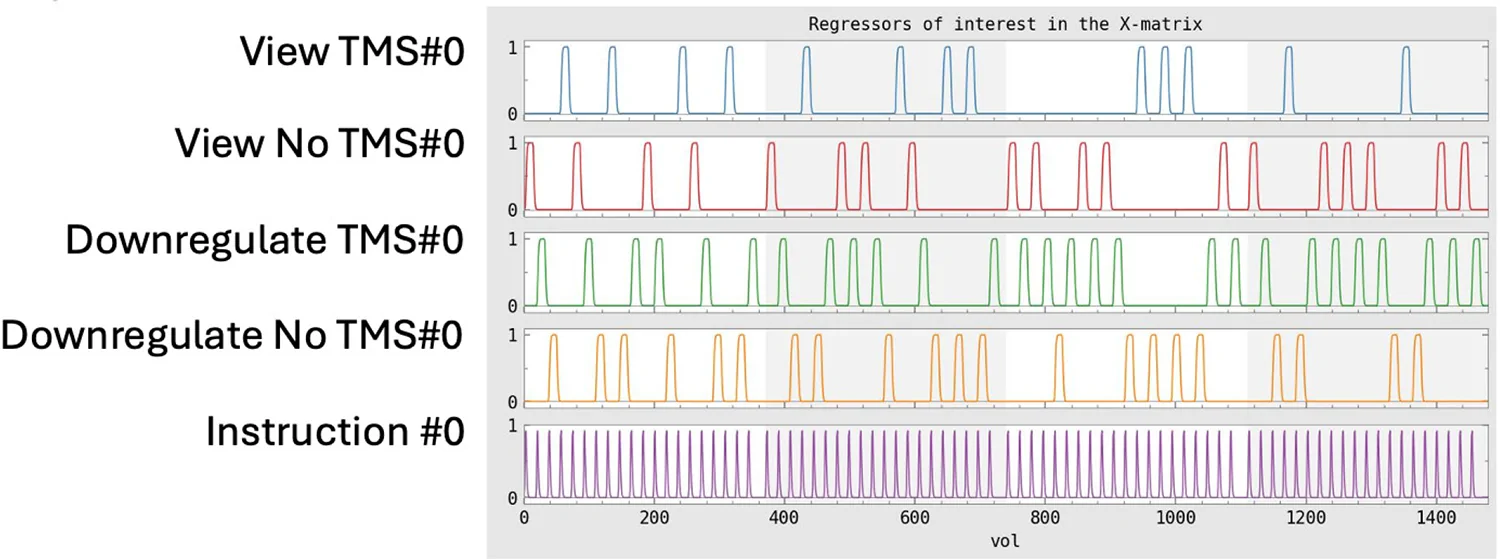
Task regressors used for offline fMRI processing.

As expected, the rTMS pulses induced a large artifact on the rt-fMRI-nf acquisition (Figure 11). As it was applied during two TRs, we opted for correcting this artifact by smoothing the signal and replacing each time point during which rTMS was applied with the average of +/− 3TRs around it. We then performed the skull stripping and estimating nonlinear alignment (via *SSwarper2*^28^) of the anatomical image from native space to MNI stereotaxic space. The *afni_proc.py* script was then used to set up a full pipeline for the fMRI analysis of each participant, including the automatic generation of a quality control HTML for evaluating the data and processing steps. The first 10 TRs (20 seconds) were excluded from analyses to allow stabilization of the baseline time series signal. All images were corrected by registration to the minimum outlier volume and spatially smoothed with a 4 mm full-width-half-maximum smoothing kernel. EPI-anatomical alignment was be performed using the lpc+ZZ cost function with local EPI unifizing for additional stability, and these datasets were checked for left/right consistency.^31^ To reduce effects of participant motion, volumes with large motion (Enorm > 0.3 mm between successive time points) were censored. Echoes were optimally combined and denoised using OC_B method. For the regression part, a multi-BLOCK regression basis was used. Separate events were modeled for the instruction (2 seconds duration), and for each block of images (24 seconds duration) as follow: Decrease, No rTMS; Decrease, rTMS; View, No rTMS; View, rTMS (Figure 7).

### 4. PRELIMINARY RESULTS, CHALLENGES, AND POTENTIAL SOLUTIONS

#### 4.1. VISIT 1: EMOTION MATCHING TASK

**Figure 8.**
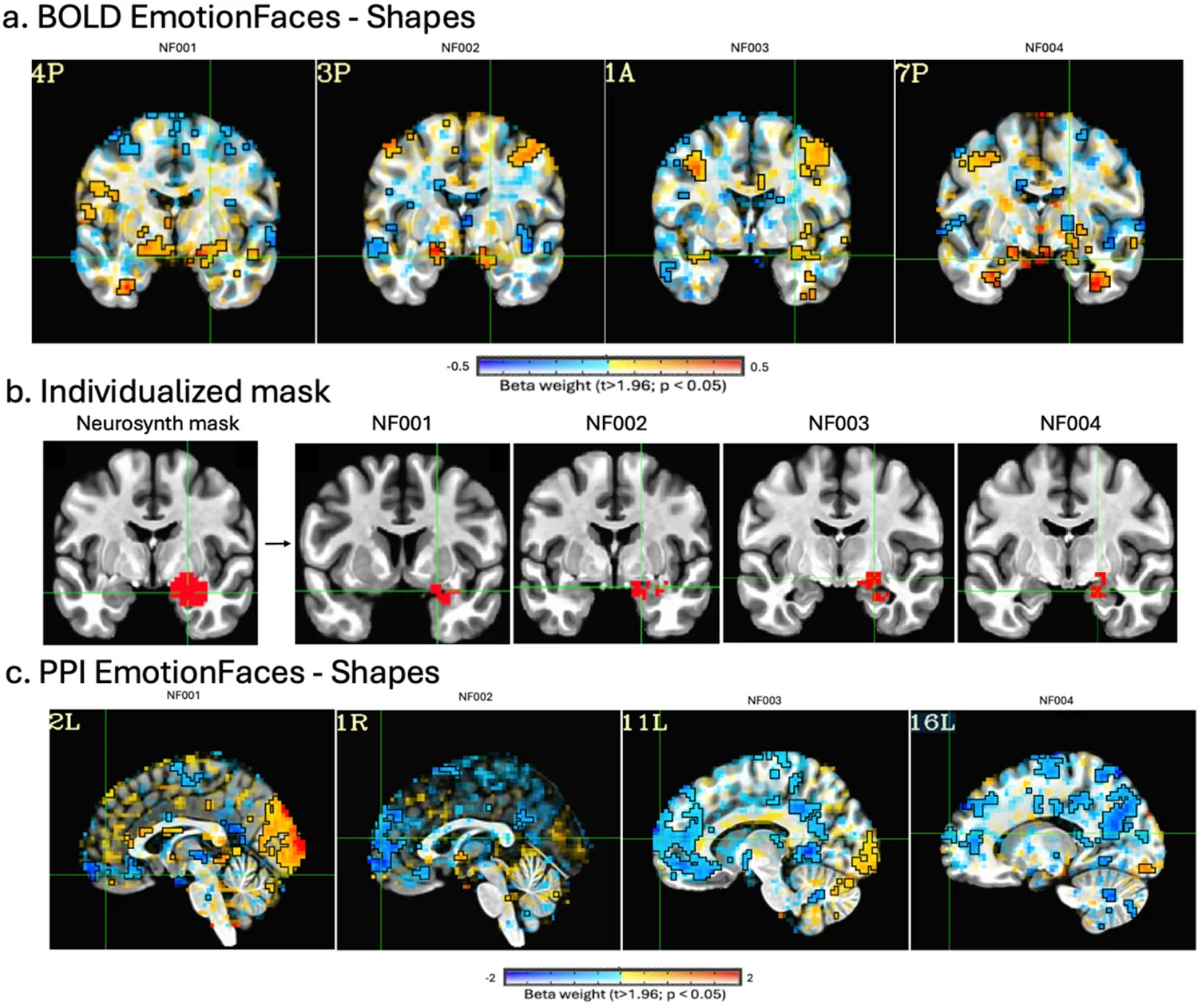
**a.** BOLD activity in the Emotional Faces - Shapes contrast; **b**. Individualized right amygdala mask for each participant, using the top 25% of activated voxel multiplied by a mask from Neurosynth; **c.** Functional connectivity between the right amygdala and the rest of the brain in the same Emotional Faces - Shapes contrast. Here and below, statistical modeling results are presented with beta weight as overlay colors,^37^ and thresholding of the statistical volume is performed transparently^38–40^; these approaches preserve context, reduce biases and enhance understanding of the results.^41,42^

All participants performed the task with high accuracy (> 90% for each participant). As expected, the comparison between emotional faces and shapes in the emotion-matching task elicited significant amygdala activation in each participant (Figure 8a), with slightly different activation loci across individuals, resulting in individualized amygdala masks (Figure 8b). The masks were used as seeds for the PPI analysis and revealed, as expected, strong negative connectivity from between the individualized amygdala loci to the medial prefrontal cortex (Figure 8c).

#### 4.2. VISIT 2–5: RT-FMRI-NF AND RTMS

##### 4.2.1. FEASIBILITY AND TOLERABILITY

No significant adverse events occurred, and all participants successfully completed the study, suggesting our experimental design is feasible. One participant requested to stop a study session after the first block of rt-fMRI-nf as they were experiencing discomfort in their neck due to the pressure from the coil when placed over the forehead. At their next session, we added more padding and they were able to complete the session without issue.

Regarding the stimulation intensity, as described in Section 2.3.1, participants had high rMTs. Achieving the intended 120% rMT required setting the device to its maximum (100% MSO), which some participant found uncomfortable, especially when applied to the mPFC. Consequently, we slowly decreased the stimulation intensity from 100% MSO or the previous visits stimulation intensity until the subject reported it was consistently tolerable. Therefore, stimulation intensity was constrained both by device limitations and participant tolerability, which (1) created an asymmetry across stimulation sites, which prevent directly comparison and (2) might limit this applica bility to this approach to subthreshold stimulation or participants with low motor threshold.

During the debriefing session, participants mentioned they were not distracted by the rTMS pulses and that they could still look at the pictures during the stimulation. They mentioned that they did not have the impression that rTMS modified their ability to decrease the height of the bar. However, three of them mentioned that they focused on their breathing to modulate the height of the bar which could lead to some bias in our results. Future studies might want to instruct participants to not use this strategy.

##### 4.2.2. AMYGDALA ACTIVATION DURING NEUROFEEDBACK TASK

**Figure 9.**
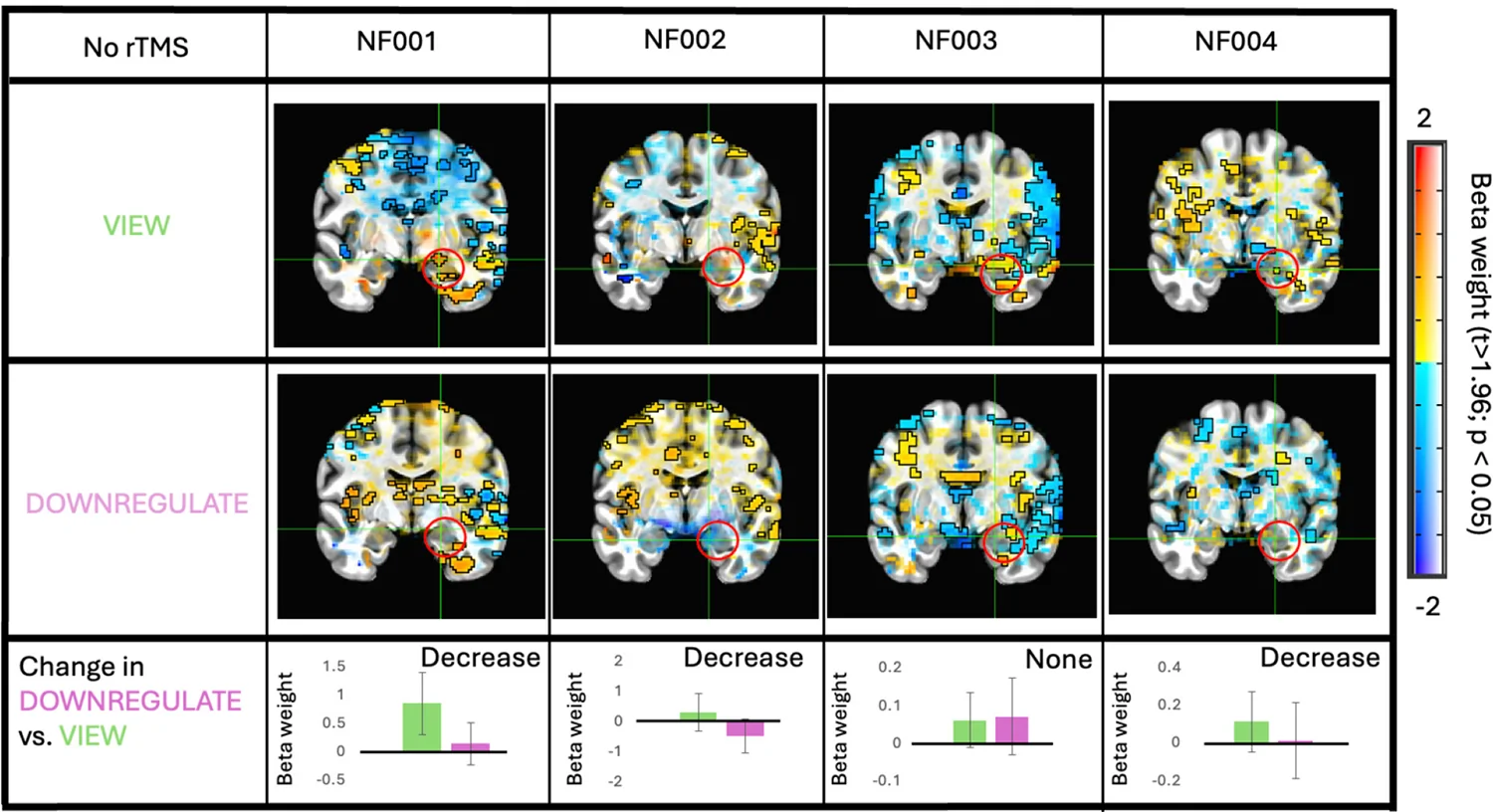
BOLD results for each participant in the View and Downregulate condition for trials obtained when the rTMS coil was placed on the vertex but not stimulating. The red circle indicates the location of the right amygdala. The ‘change in Downregulate vs. View’ row shows the beta coefficient, quantifying the difference in right amygdala activity in the Downregulate (magenta) and View (green) conditions. A decrease in activation was expected and observed in three of the participants.

For trials without concurrent rTMS, intended to isolate task-related amygdala modulation driven by neurofeedback alone, we extracted the beta coefficient in the right amygdala and demonstrated that all participants has positive amygdala activation during the View trials; and that three of them demonstrated the expected decrease in amygdala activation during Downregulate trials compared to View trials. In contrast, one participant (NF003) failed to show this differentiation, with no difference between the two conditions. This pattern indicates that volitional modulation of amygdala activity was evident in a subset of participants but was not consistently expressed across the sample, which is consistent known interindividual variability in neurofeedback performance^43^ (Figure 9).

##### 4.2.3. AMYGDALA ACTIVATION DURING NEUROFEEDBACK TASK COMBINED WITH RTMS

For trials during which rTMS was applied, substantial susceptibility-related artifacts were observed in the amygdala despite the use of interpolation procedures. To mitigate these effects, the multiband acceleration factor was removed in subsequent visits, which resulted in a qualitative reduction of artifacts, although residual signal instability remained.

**Figure 10.**
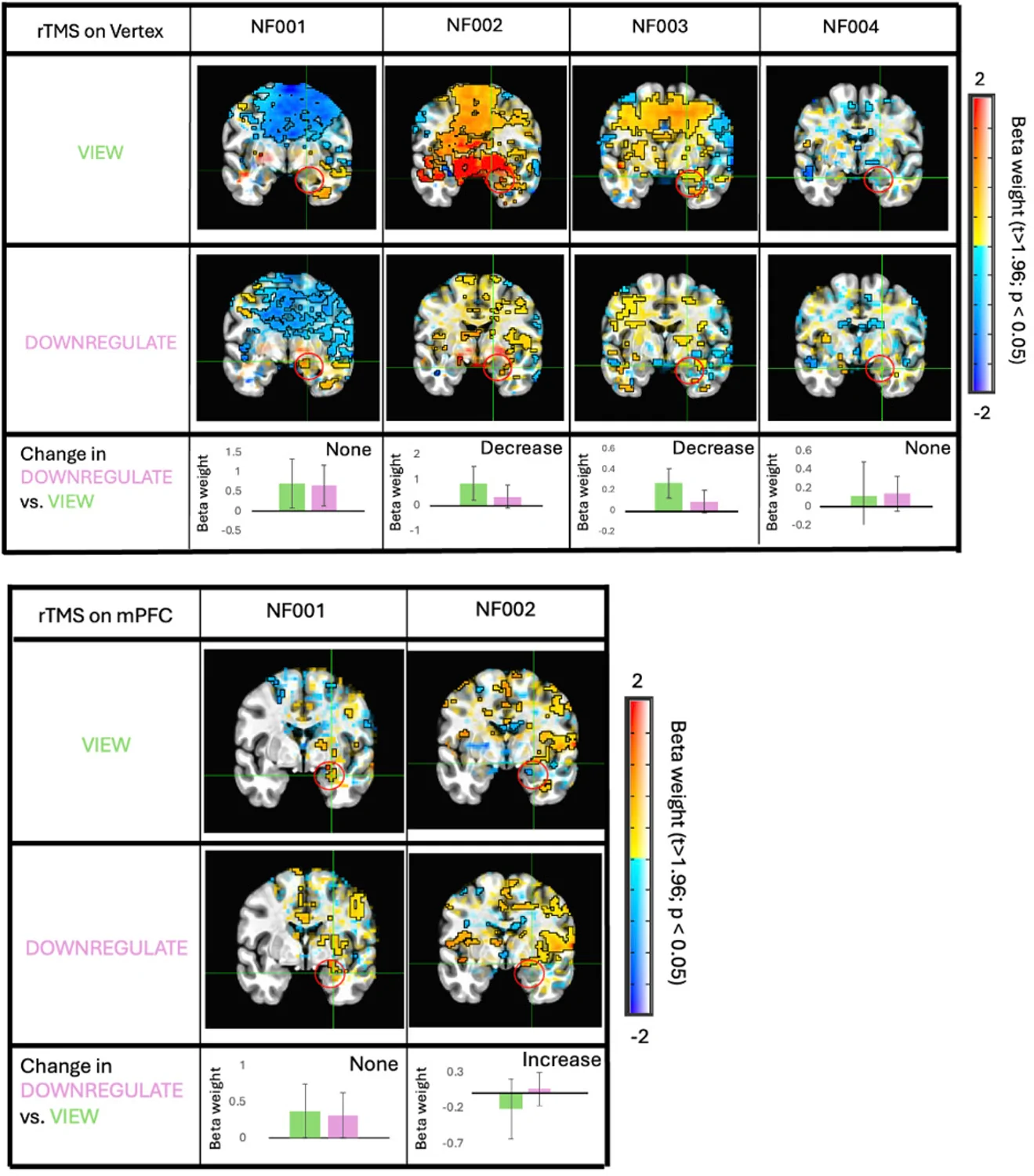
BOLD results for each participant in each of the condition of interest View and Downregulate when rTMS was applied **a.** over the vertex and **b.** over the mPFC. The red circle indicates the location of the right amygdala. The ‘change in Downregulate vs. View’ row shows the beta coefficient, quantifying the difference in the right amygdala activity in the Downregulate (magenta) and View (green) conditions. (Note that NF003 and NF004 did not receive rTMS over the mPFC as their data were used to compare the signal to noise ratio between the 7-channel and the 64-channel coil, see section 3.5). (See supplement material for last two visits for participants NF001 and NF002).

When rTMS was applied over the vertex (Figure 10a), a control condition not expected to modulate amygdala activity, two participants (NF002 and NF003) showed decreased activation during Downregulate trials compared to View trials, and the other two participants (NF001 and NF004) showed no discernible change between conditions. Therefore, amygdala activation during those trials were highly different from when no rTMS was applied.

Similarly, when rTMS was applied over the mPFC (Figure 10b), a condition hypothesized to reduce right amygdala activity during Downregulate trials, no consistent changes were observed. Indeed, one participant (NF001) did not show any substantial change between Downregulate and View trials, while the other participant (NF002) showed an increase between conditions.

Taken together, these findings demonstrate substantial inter-individual variability in amygdala BOLD responses during rTMS conditions, compounded by persistent signal artifacts in this region. As a result, no coherent or condition-specific pattern of amygdala modulation attributable to rTMS could be reliably identified in this sample.

#### 4.3. CHALLENGES AND POTENTIAL SOLUTIONS FOR FUTURE STUDIES

##### 4.3.1. RTMS COIL OVERHEATING

During the initial visits of the study, a rapid increase in rTMS coil temperature was observed when stimulating at the required high MSO intensities. Temperature increases of up to 12° C were observed by the end of an experimental block, quickly approaching the maximum safe operational temperature of the stimulator. This problem was solved by turning on the MRI fan at the end of each block and waiting for the coil temperature to cool down before starting the new block, and by increasing the flow of medical air to a nozzle on the rTMS coil see Table 2 for temperature and waiting time for each run and each participant, when recorded.

##### 4.3.2. LIMITS TO MULTI ECHO ACQUISITION DURING NEUROFEEDBACK

While our goal was to match the parameters used in Visit 1, the computational overhead to generate and export DICOM image data (to be read by AFNI’s utilities) made acquiring three echoes of EPI fMRI data not feasible for a real-time neurofeedback experiment. Indeed, the data transfer time for the 3-echo acquisition was lagged by 6 TRs (12 seconds) during most of the acquisition and increased to 8 TRs (16 seconds) toward its. Data acquisition was pared back to 2 echoes to allow its import into AFNI, along with computation of participants’ response and feedback cues, to better align temporally, as the data transfer time remained constant at 1 TR throughout the run. We are assessing other options for exporting and accessing image data in real-time.

##### 4.3.3. LIMITS TO RTMS COIL POSITIONING WITHIN A 7-CHANNEL MRI COIL

An important factor when using rTMS is ensuring that the rTMS coil is correctly positioned on the desired target. For the vertex, the coil was positioned over the midline at the posterior aspect of the head, with the center of the coil aligned to approximate the standard vertex location (Cz). For mPFC stimulation, the target was defined outside the scanner using neuronavigation, as the spot showing the strongest negative connectivity with the amygdala, as defined by the PPI analysis. In the absence of an MRI compatible neuronavigation system inside the scanner, this spot was marked on a swim cap prior to entering the scanner and served as the intended stimulation site during MRI acquisition. However, we faced physical limitations as the round MRI coil limited the placement of the rTMS coil as it was resting on the participant’s nose causing discomfort and blocking their field of view. Therefore, the TMS coil was moved to ensure participant comfort resulting in a close but not exact positioning. Because the final coil position was not tracked inside the scanner, the displacement between the intended neuronavigated target and the actual stimulation location could not be quantified. Future studies should consider the use of MRI-compatible neuronavigation or MRI-visible fiducial markers (e.g., vitamin E capsules) to permit retrospective localization of the stimulation site. Besides, the 7-channel MRI coil may not be ideal for mPFC stimulation and future studies might want to use an MRI-compatible neuronavigation system to ensure accurate coil positioning over the target, as well as the new flexible RF coil,^44^ which has a more conformable design which may reduce spatial constraints around the forehead and facilitate more precise TMS coil placement.^44^

##### 4.3.4. RTMS ARTIFACT DURATION

**Figure 11.**
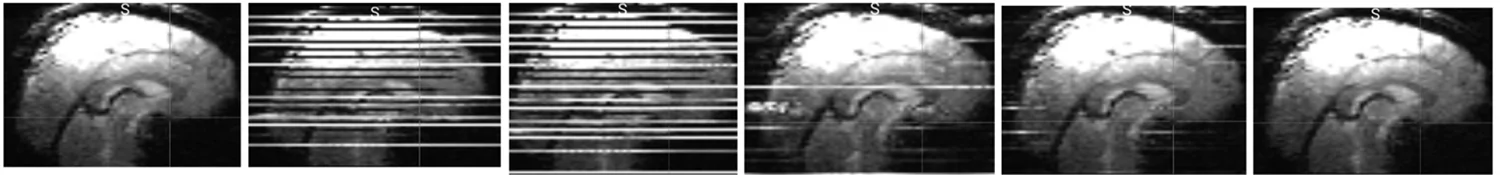
Illustration of the rTMS artifact 1TR before the pulse, during the rTMS pulse and 3 TRs after the pulse

While rTMS was applied only during 2TRs, and while we confirmed with an oscilloscope that the last pulse was sent before the beginning of the next TR, we found that the artifact for some of the echoes and some of the participants extended to more than just 2 TRs (Fig. 11). While this could be explained by acquisition timing or sampling misalignment, the occasional extension beyond one TR suggests additional contributing factors, including sequence-dependent interactions and potential residual electromagnetic effects. Notably, this pattern was not systematic, but occurred only in some echoes and stimulation blocks, further supporting a timing-dependent rather than purely hardware-driven explanation. Importantly, prior work has demonstrated that TMS-related artifacts are not necessarily restricted to the stimulated TR. For example, Weiskopf et al.^45^ showed that concurrent TMS-fMRI can introduce artifacts due to leakage currents in the stimulation circuitry, which may extend beyond the immediate pulse. Similarly, Riddle et al.^46^ reported that TMS pulses delivered during RF excitation in EPI can produce disruptions that persist across multiple volumes. In our protocol, rTMS was delivered continuously over two full TRs, making temporal overlap with RF excitation and slice acquisition highly likely. As a result, some pulses might have occurred during sensitive phases of the sequence, increasing the probability of artifacts extending into subsequent TRs. In addition, the use of multi-echo EPI increases the duration of signal acquisition within each TR, thereby extending the temporal window during which the MRI signal is susceptible to TMS-induced interference. This may further increase the likelihood of pulse–acquisition overlap and contribute to variability across echoes. To our knowledge, the specific interaction between TMS-induced artifacts and multi-echo acquisitions has not been systematically characterized, and future work comparing single- and multi-echo sequences could help clarify this effect. For this study, it suggests that our interpolation approach (section 3.3 in the method section) was not optimal to get rid of the artifact and future studies might want to stimulate in between slices to reduce these artifacts as suggested by Jackson et al.^47^

##### 4.3.5. SIGNAL TO NOISE RATIO

**Figure 12.**
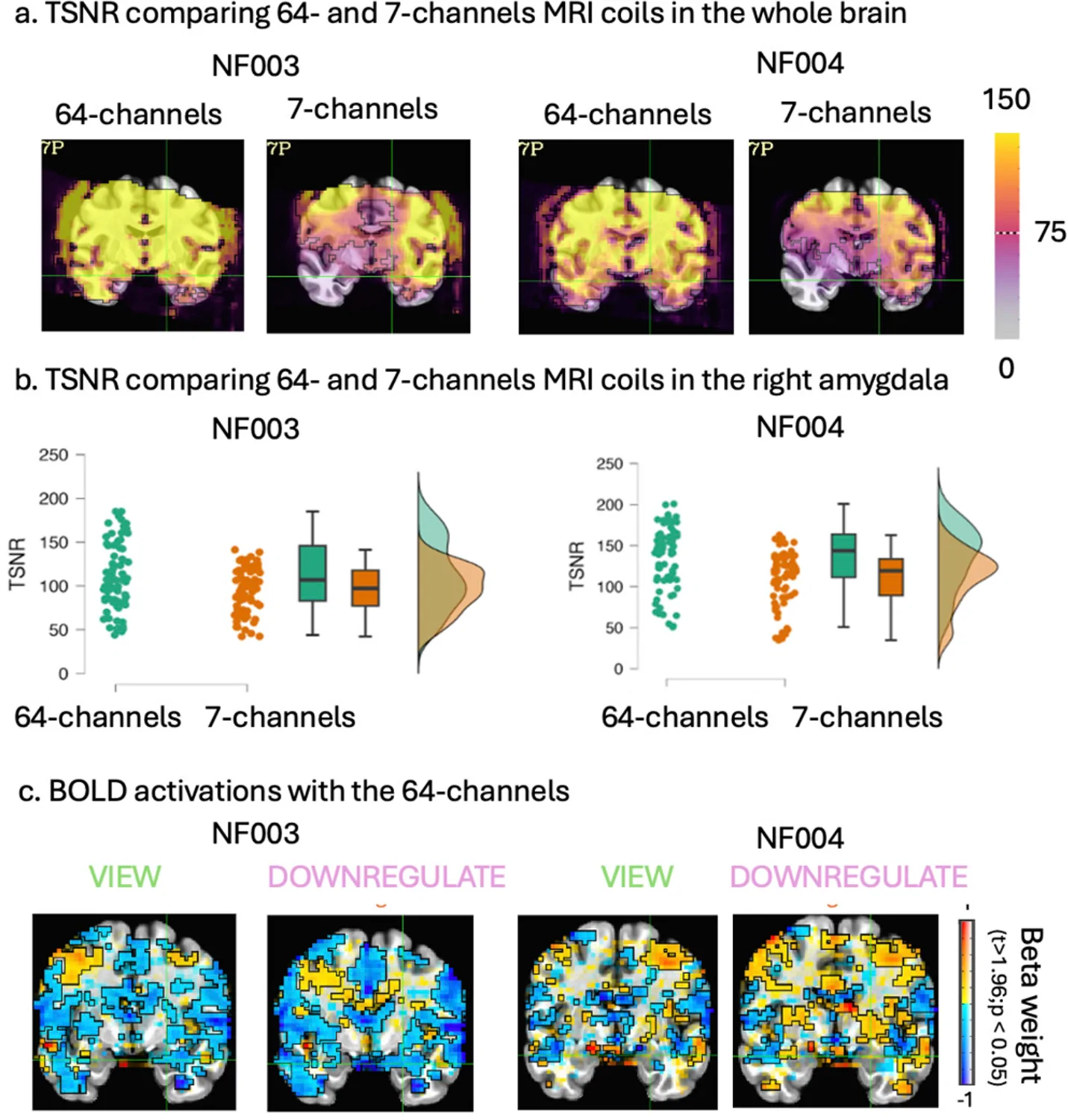
**a.** Whole brain Whole brain map of the post analysis TSNR using transparent thresholding. The boundary value is useful for showing reasonable signal strength across the region of interest and throughout much of the brain. **b.** Post analysis TSNR in the right amygdala for the 64-channel (green) and the 7-channel (orange) MRI coils showing a significantly greater signal with the 64-channel coil. **c.** BOLD activations in the View and Downregulate trials for NF003 and NF004 who performed rt-fMRI-nf without rTMS and with the 64-channel MRI coil.

To test whether the poor signal observed in Figure 9 could be due to the 7-channel MRI coil itself and the potential low resolution in deep brain regions, the last two participants (NF003 and NF004) were asked to perform the neurofeedback task without rTMS and with the 64-channel coil; and with rTMS applied over the vertex, and the 7-channel during the third visit to allow for direct comparisons between the two setups. As expected, we found a significantly better signal in the right amygdala with the 64 channels than with the 7 channels (NF003 = 111.33 +/− 39.15 vs. 95.26 +/− 25.55 t(72) = 2.98; p = 0.004; NF004 = 135.51 +/− 38.51 vs. 109.83 +/− 33.06; t(72) = 14.05; p < 0.001) (Figure 12a and 12b). However, contrary to expectations, the activations during View and Downregulate did not follow the expected pattern with no activations for the two participants during the View trials (Figure 12c). Interestingly, we reminded participants to not use deep breathing as a strategy to control the height of the bar for the last visit, and their amygdala activations were better. Future studies may benefit from pre-selection of their candidates based on the following criteria: appropriate amygdala activation with expected capacity for downregulation, ability to avoid deep breathing during trials, and absence of personality traits that interfere with neurofeedback participation.

## DISCUSSION

We present the first reported attempt to combine two technically demanding neuromodulation approaches: concurrent connectivity-based rTMS–fMRI and real-time fMRI neurofeedback, with the goal of modulating amygdala activity beyond what each method can achieve alone. While our results demonstrate the feasibility of this combined approach, they also highlight that successful integration requires substantial technical optimization.

Additionally, we demonstrated that the rTMS-related artifact extends beyond the duration of stimulation itself. Delivering stimulation between EPI slices may help mitigate these effects and yield cleaner signals.^47,48^ Systematic reporting of raw data across studies may be critical for resolving these methodological questions and advancing concurrent rTMS-fMRI research.

Then, we discussed limitations in the use of the 7-channel RF coil for stimulating the medial prefrontal cortex. Participants reported discomfort and obstructed field of view, and with the relatively low signal-to-noise ratio in the amygdala, we suggest this coil may not be optimal for targeting deeper brain structures through their connectivity with the mPFC. Future studies may benefit from focusing on more superficial regions or from using alternative hardware, such as the upcoming flexible RF coil.^44^ However, even when using a higher-resolution coil (64-channel), we did not observe the expected amygdala activation in response to aversive picture presentation. Although this task was based on prior literature^35^ and our dynamic presentation paradigm (six images presented for 4 seconds each) has been suggested to reduce amygdala habituation compared to static presentation (one image for 24 seconds),^26^ substantial inter-individual variability may have contributed to these mixed findings. Another potential explanation for the heterogeneous amygdala activation observed, even during no-TMS trials, is contamination from preceding TMS trials, possibly due to rTMS carryover effects.. Although prior work from our group has shown that short trains of 5 Hz rTMS do not affect subsequent trials,^3,8^ these findings were based solely on behavioral measures and therefore do not exclude the possibility that residual effects may have influenced underlying neural responses. In addition, as this study was designed to develop the proposed approach and demonstrate its overall feasibility in a small sample, it was not powered to assess efficacy. A larger sample size would be required to robustly detect modulation of the amygdala.

These observations underscore the importance of greater transparency in reporting. Specifically, future studies would benefit from sharing raw, individual-level data in addition to group-level results, allowing the field to better characterize variability and collectively refine experimental approaches.

## CONCLUSION

Despite these challenges, the present work represents an important step toward the integration of multimodal neuromodulation and neuroimaging approaches. Combining connectivity-based rTMS with real-time fMRI neurofeedback allows for both more thorough investigations into mechanistic neuroscience, in which we can directly test causal relationships between network activity and behavior, and develop more effective neuromodulation protocols that control for brain state and individual variability in network response.

As the field develops and overcomes the technical limitations, sources of variability, and hardware-related constraints identified here-in, this technology holds significant promise to improve efficacy and durability of neuromodulatory treatments, deepen our understanding of circuit-level mechanisms underlying affective disorders, and develop personalized, mechanism-driven interventions that go beyond symptom-based approaches. This approach could even be further developed by implementing closed-loop stimulation paradigms, where brain-state information derived from rt-FMRI-nf could be used to adaptively trigger rTMS in response to the ongoing neural activity. Such an approach may enhance stimulation specificity and improve therapeutic impact, as suggested by Moreno et al.^49^

## Figures and Tables

**Table 1.** Stimulation intensities for each participant per rTMS visit relative to RMT. (Note that NF003 and NF004 only completed one visit with rTMS as their data were used to compare the signal to noise ratio between the 7-channel and the 64-channel coil, see section 3.5).

Participant	rMT	Visit 2 (Vertex)	Visit 3 (mPFC)	Visit 4 (Vertex)	Visit 5 (mPFC)
NF001	90% MSO	90% MSO (100% rMT)	75% MSO (83.3% rMT)	70% MSO (77.8% rMT)	70% MSO (77.8% rMT)
NF002	84% MSO	80% MSO (95.2% rMT)	78% MSO (92.9% rMT)	84% MSO (100% rMT)	80% MSO (95.2% rMT)
Participant	rMT	Visit 3 (Vertex)			
NF003	90% MSO	100% MSO (111.1% rMT)			
NF004	93% MSO	100% MSO (107.5% rMT)			

**Table 2.** Coil temperature (in degree Celsius) at the beginning (Init) and end (Final) of each fMRI run as well as the waiting time between runs (Break) for each visit and each participant, when collected. Minus sign indicates that MRI was not collected, while blank sign indicates that temperature information were not recorded.

		Run 1		Break (min)	Run 2		Break (min)	Run 3		Break (min)	Run 4	
Participant	Visit	Init	Final		Init	Final		Init	Final		Init	Final
**NF001**	Visit 2	23	35	8	31	40	8	35	42			
	Visit 3	21	30	–	–	–	–	–	–	–	–	–
	Visit 4			2			12			2		
	Visit 5	21	29	3	29	35	1	35	39	–	–	–
**NF002**	Visit 2			3		41	8	36	42	9		
	Visit 3			14			7			9		
	Visit 4	21	27	1	28	32	15	27	31	4	30	33
	Visit 5	21	25	2	25	28	1	28	29	2	29	–
**NF003**	TMS Visit	21	30	13	30	37	4	37	40	2	40	41
**NF004**	TMS Visit	23	32	13	32	37	13	32	37	3	37	42

## Data Availability

Data are publicly available on OpenNeuro: https://open-neuro.org/datasets/ds007396/versions/1.0.0
